# A real-world data analysis of tirzepatide in the FDA adverse event reporting system (FAERS) database

**DOI:** 10.3389/fphar.2024.1397029

**Published:** 2024-06-07

**Authors:** Liyuan Liu

**Affiliations:** Department of Pathology, Ganzhou People’s Hospital (The Affiliated Ganzhou Hospital of Nanchang University), Ganzhou, China

**Keywords:** tirzepatide, real-world data analysis, FDA adverse events reporting system, signal detection, pharmacovigilance, obesity

## Abstract

**Background:**

Tirzepatide, a glucose-dependent insulinotropic polypeptide (GIP) receptor and glucagon-like peptide-1 (GLP-1) receptor agonist, is indicated for chronic weight management in adults with obesity or overweight as an adjunct to a reduced-calorie diet and increased physical activity. However, the safety profile of Tirzepatide-associated adverse events requires comprehensive evaluation.

**Methods:**

The AE reports from the first quarter of 2022 to the third quarter of 2023 were selected by exploring the FDA Adverse Event Reporting System (FAERS) database. The new and unexpected potenial AE signals were detected using the disproportionality analysis, including reporting odds ratio(ROR), the proportional reporting ratio (PRR) the Bayesian confidence propagation neural network (BCPNN) and the empirical Bayes geometric mean(EBGM). Then the MedDRA was used to systematically classify the results.

**Results:**

A total of 1,904,481 case reports were obtained from 2022Q2 to 2023Q3. Forty-sixth tirzepatide-induced ADRs at the preferred terms (PTs) level are associated with 8 system organ class In addition, this study uncovered multiple anticipated ADRs, such as gastrooesophageal reflux disease, dyspepsia, and vomiting, in line with the drug labels. Moreover, unexpected and significant ADRs at PTs level, such as incorrect dose administered, injection site haemorrhage, and increased appetite, were discovered and linked to Injury, poisoning, and procedural complications, General disorders and administration site conditions, and Metabolism and nutrition disorders at the System Organ Class level.

**Conclusion:**

This study offered new perspectives on the monitoring, surveillance, and management of adverse drug reactions related to tirzepatide. The outcomes of severe adverse events and their respective detection signals, along with unexpected significant adverse event signals, are important to consider in efforts to enhance clinical medication safety when using tirzepatide.

## 1 Introduction

The body mass index (BMI), calculated by dividing an individual’s weight in kilograms by their height in meters squared, is commonly used as a standard measure for defining obesity in populations. According to the World Health Organization guidelines, individuals are categorized as overweight (BMI 25-29.99) or obese (BMI ≥30). Furthermore, obesity can be classified into three categories: class I obesity (BMI 30-34.99), class II obesity (BMI 35-39.99), and class III obesity (BMI ≥40) (Elmaleh-Sachs et al., 2023). The prevalence of obesity has seen a consistent global increase since 1975, with rates rising from 3.2% to 10.8% in men and from 6.4% to 14.9% in women by 2014. Projections indicate that by 2025, around 18% of men and 21% of women worldwide will be classified as obese (NCD Risk Factor Collaboration NCD-RisC, 2016). Additionally, nearly 42% of American adults are affected by obesity, with associated links to conditions such as type 2 diabetes mellitus, hypertension, cardiovascular issues, sleep disorders, musculoskeletal problems, and premature mortality (Elmaleh-Sachs et al., 2023). The importance of implementing effective strategies in managing obesity is highlighted by these findings, as they demonstrate that achieving and maintaining a sustained weight loss of over 10% of total body weight can reduce the risk of severe health complications related to obesity and improve quality of life. The main challenge in obesity management is maintaining weight loss (Perdomo et al., 2023). New options for preventing complications of obesity are emerging through the development of innovative medications, supported by findings from trials on dietary weight loss and bariatric surgery (Drucker, 2024).

Tirzepatide is a single molecule that combines dual agonism of glucose-dependent insulinotropic polypeptide (GIP) and glucagon-like peptide-1 (GLP-1) receptors (Syed, 2022). The incretin system plays a crucial role in regulating postprandial metabolism through the action of two important incretin peptides, GIP and GLP-1, which activate their respective receptors on islet β cells and other tissues (Campbell et al., 2023). GIP and GLP-1 are crucial for controlling blood sugar levels and have a profound impact on the development of T2DM (Nauck and Müller, 2023). GIP is involved in nutrient and energy metabolism, while GLP-1 not only stimulates insulin secretion and decreases glucagon secretion but also contributes to the deceleration of gastric emptying, reduction of appetite, and enhancement of satiety. Tirzepatide was approved by the US Food and Drug Administration (FDA) for enhancing glycaemic control in adults diagnosed with T2DM, serving as a complement to dietary adjustments and physical activities in May 2022 (Syed, 2022). As a pioneering peptide that uniquely interacts with both GIP and GLP-1 incretin receptors, tirzepatide has demonstrated unparalleled outcomes in clinical studies, showcasing its ability to promote weight loss and enhance glucose control (Dahl et al., 2022; Jastreboff et al., 2022; Garvey et al., 2023). Moreover, the FDA approved tirzepatide as an adjunct to a reduced-calorie diet and increased physical activity for chronic weight management in adults with obesity or overweight who also have at least one weight-related comorbid condition, such as hypertension, dyslipidemia, type 2 diabetes mellitus, obstructive sleep apnea, or cardiovascular disease in November 2023.

In addition to promoting weight loss and improving blood sugar control, tirzepatide has been shown to reduce the risk of cardiovascular and diabetes-related events in obese patients, making it a highly recommended medication for weight management due to its significant efficacy (Hankosky et al., 2023; 2024; Xie et al., 2023). Tirzepatide has been widely used, but it has also been associated with adverse events. The most common ones are mild to moderate gastrointestinal issues such as nausea, diarrhea, constipation, and vomiting (Aronne et al., 2024). Additionally, decreased appetite and hypoglycemia related to tirzepatide have been reported (Ludvik et al., 2021). Patients receiving tirzepatide injections may also experience injection site reactions, hypersensitivity, headache, and nasopharyngitis. Moreover, pancreatitis has been observed in patients undergoing treatment with tirzepatide across multiple randomized clinical trials (Del Prato et al., 2021; Frías et al., 2021; Wadden et al., 2023).

Due to the widespread use of tirzepatide in clinical practice, it is possible that some serious adverse events have not been fully identified. Additionally, there is a significant lack of real-world data research on adverse drug reaction signals linked to tirzepatide. The FDA Adverse Event Reporting System (FAERS) database provides valuable information on adverse events associated with medications and biological products, making it a crucial tool for conducting pharmacovigilance analysis. Kumar’s team identified many of the adverse event signals of the drug data through disproportionate analysis methods, which were performed in the FAERS database (Sharma and Kumar, 2022; Jain et al., 2023; Sharma et al., 2023; Javed and Kumar, 2024). By analyzing case reports from the FAERS database, researchers can gain insights from real-world data and identify ADRs that may not be listed on the drug’s label (Singh and Kamath, 2021; Wu et al., 2022; Zhao et al., 2023; 2024). In this research, a disproportionality analysis was performed on adverse events associated with tirzepatide using the FAERS database to detecting potential signals. The objective of this research was to identify and characterize unforeseen ADRs not previously listed on the drug’s label, thereby enhancing awareness and prompting additional research into the medication’s safety.

## 2 Materials and methods

### 2.1 Data source and process

The FAERS database, an open-access post-marketing safety surveillance database, was maintained by the FDA. This database collects information on adverse events and side effects related to various medical products, including prescription and over-the-counter drugs, biological products, medical devices, and dietary supplements.

In this study, The adverse events data related to tirzepatide/Zepbound from the FDA Adverse Event Reporting System (FAERS) were collected. The data underwent collection and preprocessing using SAS and MYSQL software to ensure accuracy and consistency. The collected data involving tirzepatide in FAERS has undergone the removal of duplicated case records through individual safety report (ISR) coding. Additionally, the drug names have been mapped to RxNorm concepts and adverse drug reaction (ADR) outcomes to Medical Dictionary for Regulatory Activities (MedDRA^®^) concepts (Liu et al., 2005). To assess the safety of tirzepatide in the post-marketing period, this study conducted queries in the FAERS database to extract all AEs reported between the 2022Q2 (after FDA approval of tirzepatide) and 2023Q3. The reported role of drugs in adverse events included categories such as primary suspect (PS), secondary suspect (SS), concomitant medications (C), and interacting drugs (I). Moreover, this study categorized serious clinical outcomes such as death, life-threatening, disability, hospitalization, congenital anomalies, and required intervention to prevent permanent impairment or damage. In addition to analyzing AEs, demographic factors such as gender and age, geographical locations of reported events by continent and country, as well as indications for tirzepatide use were also taken into consideration. These supplementary indicators provide a comprehensive overview of the safety profile and potential risks associated with tirzepatide in real-world post-marketing settings.

### 2.2 Data mining algorithm

A disproportionality analysis is a crucial analytical tool within pharmacovigilance, essential for comparing the occurrence of adverse events specifically related to a study drug in comparison to all other drugs. In this study, four distinct algorithms were utilized to effectively measure the signals of tirzepatide-associated adverse events. These algorithms included the Reporting Odds Ratio (ROR), the Proportional Reporting Ratio (PRR), the Bayesian Confidence Propagation Neural Network (BCPNN), and the Empirical Bayesian Geometric Mean (EBGM) (Bate et al., 1998; Dumouchel, 1999; Evans et al., 2001; Rothman et al., 2004). The utilization of these algorithms were fundamental in successfully identifying and quantifying signals of tirzepatide-associated adverse events in the context of pharmacovigilance. To confirm the robustness of the ADRs findings, it was essential to align them with the stringent selection criteria of all four pharmacovigilance algorithms (ROR, PRR, BCPNN, and EBGM). The prerequisites were delineated in Supplementary Table S1 and the statistical algorthms including ROR, PRR, BCPNN, and EBGM are outlined below:

1) ROR Method

$$ROR=\frac{\text{ad}}{\text{bc}}$$

$$95\%CI=e^{\ln\left(ROR\right)\pm \sqrt{1.96\left(\frac{1}{a}+\frac{1}{b}+\frac{1}{c}+\frac{1}{d}\right)}}$$

Positive signal detection criteria: the lower limit of 95% CI > 1, N ≥ 3;

2) PRR Method

$$PRR=\left[a\left(c+d\right)\right]/\left[c\left(a+b\right)\right]$$

$$\chi 2=\frac{{\left(\text{ad}-\text{bc}\right)}^{2}\left(a+b+c+d\right)}{\left(a+b\right)\left(a+c\right)\left(b+d\right)\left(c+d\right)}$$

Positive signal detection criteria: PRR ≥2, χ2 ≥ 4, N ≥ 3, and *p* < 0.05;

3) BCPNN Method

$$IC=\log_{2}\frac{a\left(a+b+c+d\right)}{\left(a+b\right)\left(a+c\right)}$$

$$95\%CI=E\left(IC\right)\pm 2\times \sqrt{V\left(IC\right)}$$

Positive signal detection criteria: IC025 > 0 (IC025 denotes the lower bound of 95% CI);

4) EBGM Method

$$EBGM=\frac{aN}{\left(a+b\right)\left(a+c\right)}$$

$$95\%CI=e^{\ln\left(EGBM\right)\pm \sqrt{1.96\left(\frac{1}{a}+\frac{1}{b}+\frac{1}{c}+\frac{1}{d}\right)}}$$

Positive signal detection criteria: EBGM05 > 2 (EBGM05 denotes the lower bound of 95% CI).

## 3 Results

### 3.1 Clinical characteristics

A total of 1,904,481 case reports were obtained from the FAERS database over the span of the research period (from the 2022Q2 to 2023Q3). There were 20,043 case reports associated with tirzepatide as the primary suspected (PS). Additionally, the drug role counts of PS, SS, C, and I drug role are 20,043, 7780, 229, and 16 respectively. The detailed clinical features of events involving tirzepatide were outlined in Table 1.

**TABLE 1:** Clinical characteristics of reports associated with tirzepatide from the FAERS database (2022Q2 to 2023Q3).

	tirzepatide	
Characteristics	Counts (n)	Percentage(%)
Number of events	20,043	
Gender		
Male	3,488	17.40
Female	13,613	67.92
Unknown	2,942	14.68
Age		
<18	3	0.01
18–29	339	1.69
30–39	1,178	5.88
40–49	1870	9.33
50–59	2,106	10.51
60–69	1,205	6.01
70–79	449	2.24
80–89	53	0.26
≥90	1	0.00
Unknown	12,839	64.06
Reported Continents		
North America	19,891	99.24
Asia	141	0.70
Europe	7	0.03
South America	4	0.02
Reported Countries (Top five)		
US(United States)	19,888	99.23
JP(Japan)	69	0.34
AE(United Arab Emirates)	57	0.28
SA(Saudi Arabia)	11	0.05
BR(Brazil)	3	0.01
Indications (Top ten)		
Product used for unknown indication	11,996	59.85
Type 2 diabetes mellitus	4,396	21.93
Weight decreased	1849	9.23
Diabetes mellitus	1,094	5.46
Glucose tolerance impaired	991	4.94
Insulin resistance	309	1.54
Obesity	244	1.22
Polycystic ovaries	178	0.89
Glycosylated haemoglobin increased	161	0.80
Blood glucose increased	92	0.46
Serious Outcomes		
Death	49	0.24
Life-threatening	34	0.17
Disability	24	0.12
Hospitalization	590	2.94
Congenital anomaly	1	0.00
Required intervention to prevent permanent impairment/damage	19	0.09
Other serious medical events	756	3.77
Reporting quarter		
2022Q2^a^	4	0.02
2022Q3	1,198	5.98
2022Q4	3,628	18.10
2023Q1	4,686	23.38
2023Q2	5,077	25.33
2023Q3	5,450	27.19

^a^ Indicates the second quarter of 2022.

Regarding the case reports that documented all AEs, females (67.92%) accounted for a larger proportion than males (17.40%). Among the cases where the age of the patient was recorded, individuals aged 50 to 59 were more likely to experience AEs than other age groups, accounting for 10.51% (2,106) of cases. The majority of adverse event reports associated with tirzepatide originated from North America (99.24%), with a smaller number from Asia (0.70%), Europe (0.03%), and South America (0.02%). In terms of usage, the United States (19,888, 99.23%), Japan (69, 0.34%), United Arab Emirates (57, 0.28%), Saudi Arabia (11, 0.05%), and Brazil (3, 0.01%) were the top five countries. The most common indications for AEs related to tirzepatide were product used for unknown indication (11,996, 59.85%), type 2 diabetes mellitus (4396, 21.93%), weight decreased (1849, 9.23%), diabetes mellitus (1,094, 5.46%), and glucose tolerance impaired (991, 4.94%). Among the recorded AEs, the most common serious outcome was hospitalization (590, 2.94%), followed by death (49, 0.24%), life-threatening (34, 0.17%), disability (24, 0.12%), required intervention to prevent permanent impairment or damage (19, 0.09%), and congenital anomaly (1, 0.00%). With the exception of 0.02% reported in the second quarter of 2022, the reporting times for these adverse events were most frequently in the 2023Q3 (5,450, 27.19%), followed by the 2023Q2 (5,077, 25.33%), 2023Q1 (4,686, 23.38%), 2022Q4 (3,628, 18.10%), and 2022Q3 (1,198, 5.98%), respectively.

### 3.2 Signal detection at the system organ class (SOC) level

Signal strengths of reports of tirzepatide at the SOC level are described in Table 2. The top three SOCs ranked by case numbers were injury, poisoning and procedural complication (case numbers: 10,634, ROR (95% CI) = 12.69 (12.34–13.05), PRR (95% CI) = 6.49 (6.4–6.58), IC (IC025) = 2.62 (2.54), EBGM (EBGM05) = 6.13 (5.96)), general disorders and administration site conditions (case numbers: 7,851, ROR (95% CI) = 7.62 (7.41–7.85), PRR (95%CI) = 5.03 (4.94–5.12), IC (IC025) = 2.27 (2.18), EBGM (EBGM05) = 4.82 (4.69)), and gastrointestinal disorders (case numbers: 5,759, ROR (95% CI) = 7.79 (7.55–8.04), PRR (95% CI) = 5.84 (5.71–5.98), IC (IC025) = 2.47 (2.37), EBGM (EBGM05) = 5.56 (5.39)).

**TABLE 2:** Signal strength of reports associated with tirzepatide at the System Organ Class (SOC) level in the FAERS database.

System organ class (SOC)	SOC code	Case reports	ROR (95% CI)	PRR (95% CI)	χ2	*p*-Value	IC (IC025)	EBGM (EBGM05)	a value	b value	c value	d value
Injury, poisoning and procedural complications^a^	10,022,117	10,634	12.69 (12.34–13.05)	6.49 (6.4–6.58)	50,542.75	0.00E+00	2.62 (2.54)	6.13 (5.96)	10,634	9,409	154,120	1,730,318
General disorders and administration site conditions	10,018,065	7,851	7.62 (7.41–7.85)	5.03 (4.94–5.12)	26,175.61	0.00E+00	2.27 (2.18)	4.82 (4.69)	7,851	12,192	146,800	1,737,638
Gastrointestinal disorders	10,017,947	5,759	7.79 (7.55–8.04)	5.84 (5.71–5.98)	22,943.94	0.00E+00	2.47 (2.37)	5.56 (5.39)	5,759	14,284	92,690	1,791,748
Metabolism and nutrition disorders	10,027,433	1809	3.38 (3.22–3.55)	3.17 (3.03–3.31)	2,675.34	0.00E+00	1.63 (1.47)	3.1 (2.95)	1809	18,234	53,677	1,830,761
Investigations	10,022,891	1,674	5.81 (5.52–6.12)	5.41 (5.16–5.67)	5,784.67	0.00E+00	2.37 (2.2)	5.17 (4.91)	1,674	18,369	29,089	1,855,349
Endocrine disorders^a^	10,014,698	69	4.28 (3.37–5.46)	4.27 (3.36–5.44)	165.64	6.63E-38	2.05 (1.25)	4.13 (3.24)	69	19,974	1,518	1,882,920
Eye disorders	10,015,919	68	9.04 (7.05–11.6)	9.02 (7.03–11.56)	442.48	3.12E-98	3.06 (2.24)	8.32 (6.48)	68	19,975	709	1,883,729
Neoplasms benign, malignant and unspecified (incl cysts and polyps)	10,029,104	37	3.53 (2.54–4.9)	3.52 (2.54–4.89)	64.41	1.01E-15	1.78 (0.72)	3.43 (2.47)	37	20,006	988	1,883,450

^a^ indicates entries that could not be found in the drug manual of tirzepatide.

Remarkably, two SOC terms were not mentioned in the drug label of tirzepatide, including injury, poisoning and procedural complication (case numbers: 10,634, ROR (95% CI) = 12.69 (12.34–13.05), PRR (95% CI) = 6.49 (6.4–6.58), IC (IC025) = 2.62 (2.54), EBGM (EBGM05) = 6.13 (5.96)), and endocrine disorders (case numbers: 69, ROR (95% CI) = 4.28 (3.37–5.46), PRR (95%CI) = 4.27 (3.36–5.44), IC (IC025) = 2.05 (1.25), EBGM (EBGM05) = 4.13 (3.24)).

### 3.3 Signal detection at the preferred terms (PTs) level

There were a total of 46 ADRs terms associated with eight SOCs. As shown in Table 3, the top five strongest signal ADRs, ranked by the EBGM05 at the PTs level, included gastrooesophageal reflux disease (case numbers: 207, EBGM05 = 24.04), dyspepsia (case numbers: 409, EBGM05 = 23.54), incorrect dose administered (case numbers: 5,752, EBGM05 = 21.71), vomiting (case numbers: 970, EBGM05 = 19.3), and blood glucose abnormal (case numbers: 183, EBGM05 = 14.14)). Moreover, the top five PTs for tirzepatide based on case numbers were incorrect dose administered (case numbers: 5,752, EBGM05 = 21.71), off label use (case numbers: 3,101, EBGM05 = 2.36), injection site pain (case numbers: 2,742, EBGM05 = 9.16), nausea (case numbers: 2,456, EBGM05 = 3.62), and injection site haemorrhage (case numbers: 1,256, EBGM05 = 13.86).

**TABLE 3:** Signal strength of reports associated with tirzepatide at the Preferred Terms (PTs) level in the FAERS database.

Preferred terms (PTs)	SOC	Case reports	ROR (95% CI)	PRR (95% CI)	χ2	*p*-Value	IC (IC025)	EBGM (EBGM05)	a value	b value	c value	d value
Gastrooesophageal reflux disease	Gastrointestinal disorders	207	40.29 (34.22–47.43)	2.97 (2.52–3.49)	5,511.58	0.00E+00	4.82 (4.3)	28.3 (24.04)	207	19,836	488	1,883,950
Dyspepsia	Gastrointestinal disorders	409	36.91 (32.9–41.4)	4.28 (3.82–4.79)	10,109.85	0.00E+00	4.72 (4.35)	26.4 (23.54)	409	19,634	1,063	1,883,375
Incorrect dose administered^a^	Injury, poisoning and procedural complications	5,752	40.4 (39.06–41.8)	29.1 (28.35–29.87)	120,715.28	0.00E+00	4.49 (4.38)	22.46 (21.71)	5,752	14,291	18,587	1,865,851
Vomiting	Gastrointestinal disorders	970	27.56 (25.63–29.64)	2.67 (2.49–2.87)	18,475.33	0.00E+00	4.38 (4.14)	20.75 (19.3)	970	19,073	3,471	1,880,967
Blood glucose abnormal	Investigations	183	20.07 (17.1–23.55)	4.27 (3.65–5.01)	2,711.41	0.00E+00	4.05 (3.53)	16.59 (14.14)	183	19,860	865	1,883,573
Injection site haemorrhage^a^	General disorders and administration site conditions	1,256	18.36 (17.26–19.53)	17.27 (16.29–18.32)	16,337.90	0.00E+00	3.88 (3.68)	14.75 (13.86)	1,256	18,787	6,836	1,877,602
Increased appetite^a^	Metabolism and nutrition disorders	202	18.85 (16.2–21.94)	10.28 (8.84–11.94)	2,820.00	0.00E+00	3.98 (3.48)	15.75 (13.53)	202	19,841	1,017	1,883,421
Inappropriate schedule of product administration^a^	Injury, poisoning and procedural complications	874	11.51 (10.71–12.36)	2.61 (2.44–2.8)	7,180.48	0.00E+00	3.32 (3.08)	9.99 (9.3)	874	19,169	7,436	1,877,002
Injection site pain	General disorders and administration site conditions	2,742	12.02 (11.52–12.54)	10.51 (10.13–10.91)	21,533.37	0.00E+00	3.26 (3.12)	9.55 (9.16)	2,742	17,301	24,527	1,859,911
Thyroid hormones increased^a^	Investigations	45	13.63 (9.97–18.64)	4.21 (3.08–5.75)	459.16	7.34E-102	3.59 (2.57)	12.01 (8.78)	45	19,998	311	1,884,127
Eructation	Gastrointestinal disorders	489	10.43 (9.49–11.47)	36.17 (32.99–39.67)	3,671.53	0.00E+00	3.22 (2.9)	9.3 (8.46)	489	19,554	4,506	1,879,932
Glycosylated haemoglobin abnormal^a^	Investigations	78	11.48 (9.07–14.53)	5.72 (4.53–7.24)	662.82	3.63E-146	3.37 (2.59)	10.31 (8.15)	78	19,965	641	1,883,797
Accidental underdose^a^	Injury, poisoning and procedural complications	280	10.22 (9.03–11.57)	18.68 (16.52–21.11)	2073.93	0.00E+00	3.2 (2.79)	9.21 (8.13)	280	19,763	2,609	1,881,829
Pancreatitis	Gastrointestinal disorders	227	10.09 (8.79–11.58)	5.87 (5.12–6.73)	1,661.59	0.00E+00	3.19 (2.73)	9.12 (7.95)	227	19,816	2,137	1,882,301
Injection site paraesthesia^a^	General disorders and administration site conditions	146	10.34 (8.71–12.28)	23.5 (19.82–27.87)	1,102.75	8.36E-242	3.23 (2.66)	9.36 (7.89)	146	19,897	1,336	1,883,102
Blood glucose increased^a^	Investigations	566	9.49 (8.7–10.36)	3.94 (3.62–4.29)	3,805.79	0.00E+00	3.09 (2.8)	8.51 (7.8)	566	19,477	5,751	1,878,687
Injection site injury^a^	General disorders and administration site conditions	288	8.03 (7.11–9.06)	39.88 (35.39–44.94)	1,611.39	0.00E+00	2.89 (2.48)	7.39 (6.55)	288	19,755	3,415	1,881,023
Diabetic retinopathy	Eye disorders	68	9.04 (7.05–11.6)	4.63 (3.61–5.94)	442.48	3.12E-98	3.06 (2.24)	8.32 (6.48)	68	19,975	709	1,883,729
Hunger^a^	General disorders and administration site conditions	219	7.92 (6.9–9.1)	19.89 (17.34–22.82)	1,209.40	5.52E-265	2.87 (2.41)	7.32 (6.37)	219	19,824	2,624	1,881,814
Product administered at inappropriate site^a^	Injury, poisoning and procedural complications	231	7.78 (6.8–8.91)	6.59 (5.77–7.53)	1,247.62	2.74E-273	2.85 (2.4)	7.2 (6.29)	231	19,812	2,819	1,881,619
Product prescribing error^a^	Injury, poisoning and procedural complications	191	6.64 (5.73–7.7)	2.6 (2.24–3.01)	847.25	2.88E-186	2.64 (2.15)	6.22 (5.37)	191	19,852	2,726	1,881,712
Counterfeit product administered^a^	Injury, poisoning and procedural complications	71	7.02 (5.51–8.94)	7.66 (6.02–9.75)	339.86	6.86E-76	2.72 (1.93)	6.58 (5.17)	71	19,972	954	1,883,484
Impaired gastric emptying^a^	Gastrointestinal disorders	188	5.92 (5.1–6.86)	11.44 (9.88–13.25)	716.22	8.90E-158	2.48 (1.99)	5.58 (4.82)	188	19,855	3,011	1,881,427
Wrong patient received product^a^	Injury, poisoning and procedural complications	134	5.94 (4.98–7.08)	5.96 (5.01–7.09)	514.49	6.68E-114	2.49 (1.91)	5.62 (4.71)	134	19,909	2,133	1,882,305
Starvation^a^	Metabolism and nutrition disorders	105	5.18 (4.25–6.3)	37.28 (30.65–45.36)	333.73	1.48E-74	2.3 (1.65)	4.94 (4.06)	105	19,938	1915	1,882,523
Abdominal rigidity^a^	Gastrointestinal disorders	55	5.56 (4.23–7.29)	4.01 (3.06–5.27)	193.46	5.59E-44	2.4 (1.51)	5.29 (4.03)	55	19,988	933	1,883,505
Feeding disorder^a^	Metabolism and nutrition disorders	196	4.75 (4.11–5.48)	5.16 (4.47–5.95)	546.52	7.18E-121	2.18 (1.7)	4.53 (3.92)	196	19,847	3,913	1,880,525
Injection site urticaria^a^	General disorders and administration site conditions	325	4.33 (3.87–4.85)	9.99 (8.94–11.15)	784.33	1.38E-172	2.05 (1.67)	4.14 (3.7)	325	19,718	7,140	1,877,298
Nausea	Gastrointestinal disorders	2,456	4.29 (4.11–4.48)	3.89 (3.75–4.04)	5,233.22	0.00E+00	1.92 (1.77)	3.78 (3.62)	2,456	17,587	59,363	1,825,075
Abdominal distension	Gastrointestinal disorders	272	4.22 (3.73–4.77)	2.45 (2.17–2.76)	631.49	2.37E-139	2.02 (1.61)	4.04 (3.58)	272	19,771	6,122	1,878,316
Abdominal pain upper	Gastrointestinal disorders	486	4.01 (3.66–4.4)	2.32 (2.12–2.54)	1,030.04	5.31E-226	1.93 (1.63)	3.82 (3.49)	486	19,557	11,596	1,872,842
Injection site bruising^a^	General disorders and administration site conditions	655	3.72 (3.44–4.03)	9.25 (8.57–9.99)	1,216.23	1.81E-266	1.82 (1.56)	3.54 (3.27)	655	19,388	16,938	1,867,500
Thyroid mass^a^	Endocrine disorders	69	4.28 (3.37–5.46)	2.83 (2.22–3.6)	165.64	6.63E-38	2.05 (1.25)	4.13 (3.24)	69	19,974	1,518	1,882,920
Blood glucose fluctuation^a^	Investigations	113	4.01 (3.32–4.84)	3.44 (2.86–4.16)	243.20	7.91E-55	1.95 (1.33)	3.87 (3.2)	113	19,930	2,663	1,881,775
Weight decreased^a^	Investigations	689	3.52 (3.26–3.81)	2.23 (2.07–2.41)	1,159.35	4.16E-254	1.74 (1.48)	3.35 (3.1)	689	19,354	18,858	1,865,580
Injection site discolouration^a^	General disorders and administration site conditions	151	3.17 (2.7–3.73)	3.65 (3.1–4.29)	215.74	7.68E-49	1.63 (1.09)	3.09 (2.62)	151	19,892	4,498	1,879,940
Food craving^a^	Metabolism and nutrition disorders	156	2.98 (2.54–3.5)	13.6 (11.61–15.94)	197.61	6.94E-45	1.54 (1.01)	2.91 (2.48)	156	19,887	4,945	1,879,493
Medullary thyroid cancer	Neoplasms benign, malignant and unspecified (incl cysts and polyps)	37	3.53 (2.54–4.9)	9.1 (6.56–12.63)	64.41	1.01E-15	1.78 (0.72)	3.43 (2.47)	37	20,006	988	1,883,450
Injection site erythema^a^	General disorders and administration site conditions	928	2.76 (2.58–2.95)	11.05 (10.37–11.78)	963.55	1.51E-211	1.39 (1.17)	2.63 (2.46)	928	19,115	32,614	1,851,824
Decreased appetite	Metabolism and nutrition disorders	847	2.68 (2.5–2.88)	3.44 (3.21–3.67)	833.70	2.55E-183	1.36 (1.13)	2.57 (2.4)	847	19,196	30,486	1,853,952
Off label use^a^	Injury, poisoning and procedural complications	3,101	2.77 (2.66–2.88)	2.5 (2.41–2.58)	2,887.96	0.00E+00	1.3 (1.17)	2.46 (2.36)	3,101	16,942	116,856	1,767,582
Appetite disorder	Metabolism and nutrition disorders	104	2.6 (2.14–3.17)	3.44 (2.83–4.18)	99.60	1.87E-23	1.35 (0.71)	2.55 (2.1)	104	19,939	3,765	1,880,673
Hypoglycaemia	Metabolism and nutrition disorders	199	2.46 (2.14–2.84)	3.99 (3.47–4.59)	167.16	3.08E-38	1.27 (0.8)	2.41 (2.1)	199	19,844	7,636	1,876,802
Injection site rash	General disorders and administration site conditions	377	2.34 (2.11–2.6)	10.09 (9.12–11.16)	278.21	1.84E-62	1.19 (0.85)	2.29 (2.06)	377	19,666	15,287	1,869,151
Injection site discomfort^a^	General disorders and administration site conditions	186	2.42 (2.09–2.8)	7 (6.05–8.09)	150.06	1.68E-34	1.25 (0.76)	2.37 (2.05)	186	19,857	7,259	1,877,179
Injection site pruritus	General disorders and administration site conditions	578	2.27 (2.09–2.47)	10.2 (9.41–11.07)	389.92	8.62E-87	1.14 (0.86)	2.21 (2.03)	578	19,465	24,326	1,860,112

^a^ indicates unexpected AEs, of tirzepatide from FAERS, database, which are not in drug label.

Among the unexpected significant ADRs revealed, the top five strongest signal ADRs ranked by the EBGM05 were incorrect dose administered (case numbers: 5752, EBGM05 = 21.71), injection site haemorrhage (case numbers: 1256, EBGM05 = 13.86), increased appetite (case numbers: 202, EBGM05 = 13.53), inappropriate schedule of product administration (case numbers: 874, EBGM05 = 9.3), and thyroid hormones increased (case numbers: 45, EBGM05 = 8.78). Additionally, the top five PTs for these unexpected significant ADRs based on case numbers were incorrect dose administered (case numbers: 5,752, EBGM05 = 21.71), off label use (case numbers: 3,101, EBGM05 = 2.36), injection site haemorrhage (case numbers: 1256, EBGM05 = 13.86), injection site erythema (case numbers: 928, EBGM05 = 2.46), and inappropriate schedule of product administration (case numbers: 874, EBGM05 = 9.3).

## 4 Discussion

This study conducted a thorough analysis of adverse drug reactions (ADRs) associated with tirzepatide using data from the FAERS database. By employing various statistical methods such as ROR, PRR, BCPNN, and EBGM, this study examined 1,904,481 case reports and identified 20,043 cases of suspected ADRs linked to tirzepatide. There was a significant predominance of female subjects, making up almost two-thirds (67.92%), while male subjects accounted for a smaller proportion of 17.40%. This observation is consistent with findings from two prominent epidemiological studies that have shown a higher prevalence of obesity among adult women compared to adult men in the United States (Abarca-Gómez et al., 2017; Phelps et al., 2024). Furthermore, among the recorded cases, individuals aged 50-59 exhibited a higher likelihood of experiencing adverse events, comprising 10.51% (2,106) of the total cases. This was followed by the 40-49 age group with 9.33% (1,870) of cases, and the 60-69 age group with 6.01% (1,205). These results underscore the importance of closely monitoring the safety of tirzepatide in middle-aged and elderly patients.

As shown in Table 1, it presented the prevalent indications mentioned in relevant case studies of tirzepatide, encompassing type 2 diabetes mellitus, weight loss, impaired glucose tolerance, insulin resistance, obesity, increased glycosylated hemoglobin, and elevated blood glucose levels. These indications were consistent with the prescribed applications of tirzepatide. Notably, polycystic ovary syndrome was mentioned as an indication in 178 case reports. Polycystic ovary syndrome, known as PCOS, is a prevalent endocrine-metabolic condition among women in their reproductive years, affecting approximately 15% of the female population. Characterized by insulin resistance, PCOS is frequently linked to obesity and type 2 diabetes. Women diagnosed with polycystic ovarian syndrome commonly exhibit a heightened risk for developing metabolic syndrome and potential cardiovascular health complications in the long run (Engmann et al., 2017; Arya et al., 2021). Furthermore, findings from a 10-year case-control study revealed that the onset of diabetes tends to occur a decade earlier in females with PCOS compared to individuals without the condition (Ng et al., 2019). Based on the aforementioned evidence, it is reasonable to consider using tirzepatide for the treatment of PCOS. Among these reported events, hospitalization emerged as the most frequent serious outcome. Furthermore, tirzepatide is linked to various serious outcomes such as death, life-threatening conditions, disability, interventions to prevent permanent damage, and congenital anomalies. The findings of this study underscore the significance of fortifying early warning systems and implementing vigilant monitoring for ADRs linked to tirzepatide.

In this research, tirzepatide was found to be linked with 46 ADRs at the PTs level across 8 distinct organ systems. Within the eight SOCs associated with tirzepatide, the category of general disorders and administration site conditions encompasses 12 ADRs, representing the highest number of ADRs among all SOCs. Apart from hunger, these ADRs principally pertain to injection site reactions, which numerous clinical trials have reported (Frías et al., 2021; Ludvik et al., 2021; Rosenstock et al., 2021; Wadden et al., 2023). These ADRs pertaining to injection sites included documented events such as injection site pain, rash, and pruritus, as described in the product instructions. Additionally, ADRs such as injection site paresthesia, discoloration, and erythema have also been observed, despite not being explicitly detailed in the instructions. The incidence of such events is strongly correlated with the weekly subcutaneous administration of tirzepatide via a single-dose pen. Simultaneously, the gastrointestinal disorders category comprises 10 ADRs, constituting the second-largest grouping of ADRs among all SOCs. Gastrooesophageal reflux disease (case numbers: 207, EBGM05 = 24.04) and dyspepsia (case numbers: 409, EBGM05 = 23.54) emerged as the top two PTs linked to tirzepatideb according to the EBGM05 rankings. Furthermore, ADRs associated with gastrointestinal disorders, such as vomiting, eructation, nausea, and upper abdominal pain, have been noted in several randomized clinical trials (Frias et al., 2020; Del Prato et al., 2021; Wadden et al., 2023). In this research, pancreatitis was identified using four distinct algorithms during the signal detection process at the PT level. Importantly, tirzepatide has not been evaluated in individuals with a history of pancreatitis, leaving it unclear whether such a history places patients at an increased risk of developing pancreatitis while on tirzepatide. Consequently, it is imperative for physicians to inform patients about the potential risk of pancreatitis associated with this medication. Doctors should advise patients to cease tirzepatide immediately and seek medical attention if they experience symptoms indicative of pancreatitis, such as severe abdominal pain radiating to the back, or vomiting. During the study, two unforeseen adverse drug reactions (ADRs), impaired gastric emptying and abdominal rigidity, were identified, although they were not included in the medication’s prescribing information. There might have a correlation exists between abdominal rigidity and tirzepatide-induced pancreatitis. This discovery underscores the necessity for heightened surveillance of ADRs, especially in patient groups receiving concurrent treatment with drugs that suppress gastrointestinal motility or provoke abdominal muscle contractions.

In accordance with literature on two randomized, open-label, parallel-group, phase 3 trials, this study discovered that patients with type 2 diabetes mellitus who use tirzepatide may be at risk for developing diabetic retinopathy (Del Prato et al., 2021; Ludvik et al., 2021). The rapid improvement in glucose control has been associated with a temporary worsening of diabetic retinopathy. Therefore, patients with a history of diabetic retinopathy should be closely monitored for any progression of the condition. Additionally, regarding ADRs related to metabolism and nutritional disorders, the tirzepatide instructions indicate potential ADRs such as decreased appetite, appetite disorder, and hypoglycemia. Nevertheless, this study also uncovered unexpected ADRs including increased appetite, food craving, starvation, and feeding disorder. It is evident that the ADRs associated with increased appetite and food cravings are in stark contrast to those characterized by decreased appetite and appetite disorders. Furthermore, there may be a potential connection between starvation and hypoglycemia. Healthcare professionals should maintain vigilance, as tirzepatide has the potential to lower blood glucose levels and precipitate hypoglycemia during diagnosis and therapy. It is imperative for physicians to advise patients of the hypoglycemic risk and to educate them regarding its indicators and manifestations. For those with diabetes mellitus, it is crucial to monitor blood glucose levels pre- and post-initiation of tirzepatide therapy. The hypoglycemic risk can be mitigated by decreasing the dosage of sulfonylurea or insulin when used in conjunction. In addition, during the signal detection at the PTs level related to the SOC of investigations, several ADRs were identified. The ADRs comprised an increase in blood glucose levels, abnormal blood glucose levels, fluctuations in blood glucose levels, and abnormalities in glycosylated hemoglobin levels. This finding highlights the importance of daily monitoring of blood glucose and related indicators in patients. Moreover, the increased ADR of thyroid hormones prompts us to consider the potential impact of tirzepatide on human hormone levels and endocrine organs and systems, including the thyroid gland.

Despite the advantages associated with conducting large-scale population studies and employing data mining techniques in this research endeavor, it is imperative to acknowledge several limitations. Firstly, the FAERS database, being an open-access spontaneous reporting system, is susceptible to the upload of incomplete and inaccurate information by various submitters. This lack of standardization in data quality introduces potential biases into the analysis. Additionally, controlling for confounding variables such as dosage, duration of use, comorbidities, and concurrent drug therapies, which may influence adverse events (AEs), poses a significant challenge. Moreover, due to the absence of comprehensive data on the total number of patients using tirzepatide in real-world settings, it is not feasible to accurately calculate the true incidence rates of each AE. Furthermore, this study’s inability to establish a causal relationship between tirzepatide and adverse drug reactions (ADRs) is noteworthy. Disproportionality analysis, while indicating the strength of a signal, merely offers statistical significance without quantifying risk or causality. Nonetheless, the wealth of international records available supports this study’s capacity to quantify potential risks associated with tirzepatide. However, it is essential to recognize that the true risk of these ADRs can only be determined through prospective studies. Therefore, while the research contributes valuable insights, further investigation is warranted to comprehensively understand the risks associated with tirzepatide usage.

## 5 Conclusion

The study utilized data from the FDA Adverse Event Reporting System (FAERS) to investigate the potential association between tirzepatide and adverse drug reactions (ADRs). The analysis identified several previously unrecognized ADRs, including elevated thyroid hormones, abdominal rigidity, and feeding disorders, that may manifest in patients using tirzepatide. It should remain vigilant for these newly identified ADRs that may be associated with tirzepatide. In summary, the findings of this study contribute valuable empirical evidence to the understanding of tirzepatide’s safety profile following its release to the market.

## Figures and Tables

**Table:** 

**FAERS**	FDA Adverse Event Reporting System
**GIP**	glucose-dependent insulinotropic polypeptide
**GLP-1**	glucagon-like peptide-1
**AEs**	adverse events
**ADRs**	adverse drug reactions
**SOC**	system organ class
**PTs**	preferred terms
**PS**	primary suspected
**BMI**	body mass index
**T2DM**	type 2 diabetes mellitus
**FDA**	the US Food and Drug Administration
**MedDRA**	Medical Dictionary for Regulatory Activities
**PS**	primary suspect drugs
**SS**	secondary suspect drugs
**C**	concomitant medications
**I**	interacting drugs
**ROR**	Reporting Odds Ratio
**PRR**	Proportional Reporting Ratio
**BCPNN**	Bayesian Confidence Propagation Neural Network
**EBGM**	Empirical Bayesian Geometric Mean
**95% CI**	95% confidence interval
**χ2**	chi-squared
**IC**	information component
**IC025**	the lower limit of 95% CI of the IC
**E(IC)**	the IC expectations
**V(IC)**	the variance of IC
**EBGM05**	the lower limit of 95% CI of EBGM
**PCOS**	polycystic ovary syndrome
**MTC**	medullary thyroid carcinoma
**MEN2**	multiple Endocrine Neoplasia syndrome type 2

## Data Availability

The original contributions presented in the study are included in the article/Supplementary Material, further inquiries can be directed to the corresponding author.
