# Supplementary material for: A real-world data analysis of tirzepatide in the FDA adverse event reporting system (FAERS) database
Source: Front Pharmacol. 2024 Jun 7;15:1397029. doi: 10.3389/fphar.2024.1397029 (PMC11190169; doi:10.3389/fphar.2024.1397029)
Supplement: Supplementary file 1 [file Table1.DOCX]

Supplementary Material

# Supplementary Tables

| **Supplementary Table 1** Table matrix. | | |
| --- | --- | --- |
|  | Target AEs | Non-target AEs |
| tirzepatide | a | b |
| Non-tirzepatide | c | d |
| N=a+b+c+d | | |
| AEs: adverse events | | |
